# Tubercles Developed by Intermittent Fever

**Published:** 1841-08

**Authors:** 


### TUBERCLES DEVELOPED BY INTERMITTENT FEVER.

The development of tubercles, it is well known, is favoured by whatever causes impair the healthy tone of the system. Tubercular consumption, for a year or two past, has been more common than usual in some parts of Tennessee, and it is worthy of remark, that intermittent fever also prevailed in those places, to an unusual extent during the last two autumns. Visceral obstructions have attended many of these cases of intermittent, rendering the cure difficult, and where the chills have continued to recur through the winter and following spring, phthisis has been but too frequently the consequence. This, indeed, is now one of the most dreaded of the sequelæ of intermittent fever in that region of country, and increasing the necessity of arresting the disease as early as possible. In a former number we have spoken of the preparations of iron as adapted to cases of obstinate and protracted chills and fever, removing the anemic condition of the system which attends upon them; and we have now, upon the authority of some of the practitioners of Tennessee, to mention the sulphate of copper as a remedy which has been found superior to the salts of iron in this form of the disease. Y.

